# Identification of new *Corynebacterium pseudotuberculosis* antigens by immunoscreening of gene expression library

**DOI:** 10.1186/s12866-017-1110-7

**Published:** 2017-09-21

**Authors:** Cleber Eduardo Galvão, Stenio Perdigão Fragoso, Carina Elisei de Oliveira, Odinéia Forner, Renata Ribeiro Bastos Pereira, Cleber Oliveira Soares, Grácia Maria Soares Rosinha

**Affiliations:** 1 Federal University of Mato Grosso do Sul (UFMS), Campo Grande, MS Brazil; 2 Carlos Chagas Institute, Oswaldo Cruz Foundation (ICC-FIOCRUZ), Curitiba, PR Brazil; 3 Dom Bosco Catholic University (UCDB), Campo Grande, MS Brazil; 4 Federal University of Paraná (UFPR), Curitiba, PR Brazil; 5 Embrapa Beef Cattle, Animal Health - Animal Genetic Engineering Laboratory, Campo Grande, MS Brazil

**Keywords:** *Corynebacterium pseudotuberculosis*, Caseous lymphadenitis, Sheep, Gene expression, Library

## Abstract

**Background:**

Caseous lymphadenitis (CLA) is a disease that affects sheep, goats and occasionally humans. The etiologic agent is the *Corynebacterium pseudotuberculosis* bacillus. The objective of this study was to build a gene expression library from *C. pseudotuberculosis* and use immunoscreening to identify genes that encode potential antigenic proteins for the development of DNA and subunit vaccines against CLA.

**Results:**

A wild strain of *C. pseudotuberculosis* was used for extraction and partial digestion of genomic DNA. Sequences between 1000 and 5000 base pairs (bp) were excised from the gel, purified, and the digested DNA fragments were joined to bacteriophage vector ZAP Express, packaged into phage and transfected into *Escherichia coli*. For immunoscreening a positive sheep sera pool and a negative sera pool for CLA were used. Four clones were identified that strongly reacted to sera. The clones were confirmed by polymerase chain reaction (PCR) followed by sequencing for genomic comparison of *C. pseudotuberculosis* in GenBank. The genes identified were *dak2*, *fagA*, *fagB*, NlpC/P60 protein family and LPxTG putative protein family.

**Conclusion:**

Proteins of this type can be antigenic which could aid in the development of subunit or DNA vaccines against CLA as well as in the development of serological tests for diagnosis. Immunoscreening of the gene expression library was shown to be a sensitive and efficient technique to identify probable immunodominant genes.

## Background

*Corynebacterium pseudotuberculosis* is a Gram-positive, facultative intracellular bacillus. It is the etiological agent of caseous lymphadenitis disease (CLA), which mainly affects sheep and goats. A factor that drives research for CLA is that it causes economic losses in sheep and goat production. CLA is a chronic and sometimes subclinical infection, which makes its diagnosis and management difficult. CLA pathogenesis is a slow process resulting in a chronic disease [1]. In general, infections are retained to lymph nodes, however, by the spread of exotoxin hemolytic phospholipase D (PLD) it can become systemic, causing infection of the entire lymphatic system [2].

The eradication of CLA is difficult because its diagnosis is usually based on clinical signs (visualization of superficial abscesses in lymph nodes), which generally appear at a late stage. Thus, the infected animal becomes a probable disseminator of the pathogen [3–5]. The ineffectiveness of drug therapy and the unsuccessfulness of early clinical diagnosis makes disease control difficult [6]. The standard diagnostic test is bacterial culture, followed by the identification of *C. pseudotuberculosis* with the use of biochemical tests. The material, for this bacterial culture, is collected from the abscess and cultivating it in growth medium BHI-ágar with sheep blood 5% [7].

CLA prophylaxis and control can be carried out by drainage of superficial infected lymph nodes or surgical removal thereof. Another method is vaccination of the herd. There are commercial vaccines composed of combinations of antigens against other pathogens and C. *pseudotuberculosis*, where the PLD protein, used in the same formulation, is a toxoid against CLA [8–11]. A specific vaccine formulation against CLA also exists and is composed of live naturally attenuated *C. pseudotuberculosis*. This vaccine was developed in Brazil by the Bahia Company for Agricultural Development (EBDA) and is known as 1002 (name obtained from the *C. pseudotuberculosis* 1002 strain) [12].

There are few options of vaccines for the prevention and control of CLA. Most studies which seek to develop vaccine formulations use bacterial genes (DNA vaccines) or bacterial proteins (subunit vaccines). One example is a study carried out with the gene that encodes heat shock protein 60(HSP60), a chaperonin of *C. pseudotuberculosis*, in which both DNA and subunit vaccines of this gene were tested against experimental CLA [13, 14]. Another type of vaccine formulation includes attenuated strains by gene knockout. A recent study used an attenuated strain of *C. pseudotuberculosis* in which the *ciuA* gene (gene involved in the transport iron citrate to the bacterium metabolism) was knocked out. This vaccine induced both cellular and humoral responses after challenge with the wild type strain [15].

In order to develop DNA, attenuated or subunit vaccines, studies are required in order to identify genes related to bacterial virulence. Recent studies on the *C. pseudotuberculosis* genome and its molecular determinants of virulence provide new alternatives to identify genes that encode possible antigenic proteins, that is, they are genes that can be study targets for vaccine formulations [16].

The identification of new antigens may offer benefits and potential applications in vaccine development and even the development of new diagnostic tools for the identification of *C. pseudotuberculosis* in sheep and goat samples. The discovery of antigenic properties of proteins can help in the overall understanding of pathogenesis, as well as help to understand the factors related to bacterial virulence. The aim of the present study was to identify and isolate antigens by immunoscreening from a gene expression library of *C. pseudotuberculosis*.

## Methods

### Bacterial strains

The *E. coli* strains XL1-Blue MRF’ and DH5α were purchased from Agilent technologies, INC (Santa Clara, CA, USA) and, Thermo Fisher Scientific, INC, respectively.

The wild strain of *Corynebacterium pseudotuberculosis* CBO 28033, which was used for library construction, was isolated aseptically from an external abscess from sheep by needle aspiration of closed lymph nodes. Identity was confirmed by biochemical tests (API CORYNE, Biomerieux, Marcy l’Etoile, France) for *C. pseudotuberculosis* biovar *ovis.*

### Extraction of genomic DNA from *Corynebacterium pseudotuberculosis* CBO 28033

An adapted protocol was used for genomic DNA extraction [17]. Two ml cultures of *C. pseudotuberculosis*, grown for 48 h, were centrifuged at 18514 xg for 10 min. Pellets were washed once and resuspended in 0.8 ml of TE Buffer (10 mM Tris–HCl pH 7.0, 10 mM EDTA pH 8.0) with 10.3% of glucose, 8 mg of lysozyme and left overnight at 37 °C under constant stirring. Afterwards 100 μl of 10% sodium dodecyl sulfate (SDS) and 20 μl of proteinase K were added to samples, followed by vortex mixing and incubated in a water bath at 56 °C for 5 h. Then, an equal volume of phenol was added and the tubes were stirred for 1 h in an orbital shaker. Tubes were centrifuged at 15700 xg for 15 min and supernatants were transferred to other tubes where the last step was repeated.

Later, an equal volume of phenol-chloroform was added and tubes were shaken for 10 min then centrifuged under the same previous conditions. This step was repeated again and an equal volume of phenol-chloroform: isoamyl alcohol was added to the recovered aqueous phase with the same procedures as previously described. DNA was precipitated by adding 0.6 volumes of isopropanol and the tubes were centrifuged under the same previous conditions. Subsequently, pellets were washed with 70% ethanol and dried. Pellets were resuspended in 50 μl of endonuclease free water and treated with RNase for 30 min at 37 °C. DNAs were frozen at −20 °C until usage.

The integrity of genomic DNA was estimated on a 0.8% agarose gel prepared in 1× TAE buffer and stained with ethidium bromide. The concentration was obtained by spectrophotometer (Gene Quant®), using wavelengths 230, 260 and 280 nm.

### Partial enzymatic digestion of the genomic DNA

Approximately 1 μg of genomic DNA of *C. pseudotuberculosis* CBO 28033 was used for each partial digestion reaction with the enzyme *Sau*3AI (New England Biolabs®) at 0.7 U/μl (incubated at 37 °C for 16 h in a total reaction volume of 50 μl with 1X NEBuffer 1.1). The reactions were fractionated on 0.8% agarose gel and stained with ethidium bromide. Afterwards, 1000–5000 bp DNA fragments were excised and these were purified with the QIAEX II (Qiagen®) kit.

### Construction of *Corynebacterium pseudotuberculosis* gene expression library

Purified DNA was measured and ligated to ZAP Express® Predigested Vector by the Zap Express Predigested Gigapack® III Gold Cloning Kit” (purchased from Stratagene, La Jolla, CA, USA, now Agilent Technologies, Inc.) with the BamHI/CIAP treated vector (former catalog #239615, currently not available), according to the following reaction: 1 μg of λ ZAP Express; 200 ng of insert; 0.5 μl of 10X ligase buffer; 2 U of ligase. Four microliters of the binding reaction were added to the encapsidation extract of Gigapack gold plus (Stratagene®). The material was then incubated for 2 h at room temperature, after which 500 μl of SM buffer (5.8 g NaCl, 2 g MgSO_4_.H_2_O, 50 ml Tris–HCl pH 7.5, 5 ml 2% gelatin) and 20 μl of chloroform were added. The mixture was centrifuged for debris removal and stored at 4 °C.

### Validation of *Corynebacterium pseudotuberculosis* gene expression library by PCR

Polymerase chain reaction was carried out to verify if there were DNA fragments of *C. pseudotuberculosis* in the library. For this, specific primers, for amplification of 924 bp of the *pld* gene of *C. pseudotuberculosis*, were used in a reaction containing the entire library. The primers used included: *pld*coryF (5 ‘- ATG GAG AGG AAA GTT GTT TTA - 3’) and *pld*coryR (5′- CCA TCA GTT CGG ATC CGC - 3′). These primers were designed using the Oligo® primer analysis software, with the GenBank sequence of *C. pseudotuberculosis* access CP001829.

The *pld* gene was amplified in a reaction with a final volume of 50 μl containing: 5 μl buffer (1X), 1.5 μl MgCl_2_ (1.5 mM), 2 μl *pld*coryF (foward primer) (20 pmol), 2 μl of *pld*coryR (reverse primer) (20 pmol), 1 μl of dNTP (10 mM), 37 μl of ultrapure water, 0.5 U/μl Taq DNA polymerase and 1 μl of the entire library or 1 μl of genomic DNA of *C. pseudotuberculosis* for positive control. Amplification took place in a thermocycler, with an initial temperature of 95 °C followed by 35 cycles of 95 °C for 4 min for denaturing of double stranded DNA, 55 °C for 1 min for primer annealing, 72 °C for 2 min for DNA strand extension. A final extension at 72 °C for 7 min was followed by termination at 4 °C.

### Immunoscreening of gene expression library

Sera from sheep with clinical CLA signs were selected. These sera belonged to the Embrapa Beef Cattle bank and were previously analyzed by ELISA (serological entitlement data not shown). Two sera (s.1 and s.2) with high prevalence of antibodies against *C. pseudotuberculosis* sera were selected as positive for immunoscreening. Five sera (s.3, s.4, s.5, s.6 and s.7) with average prevalence, but considered positive for CLA were selected to form a sera sample pool. Another serum (ns) without antibodies against CLA and which was obtained from an animal with no clinical signs of disease was selected as a negative control for serum immunoscreening.

Immunoscreening was carried out from a 10^−4^ dilution of the gene expression library of *C. pseudotuberculosis* (4 × 10^8^ PFU), where 100 μl of recombinant phage were incubated for 15 min at 37 °C with 150 μl of a bacterial culture of *E. coli* XL1Blue with an OD = 1. Then, 4 ml of soft agar was added and this mixture was heated to 42 °C and immediately poured on a plate of LB containing 10 mM MgSO_4_. Six lysis plates with 2 × 10^3^ PFU/plate were prepared for each positive serum and one plate was used for the negative serum control. The plates were incubated at 37 °C overnight and on the following day each plate was placed on a nitrocellulose membrane soaked in 10 mM IPTG.

To mark the membrane position, perforations with needles were performed and the plates were incubated for 4 h at 37 °C. The membranes were then removed with tweezers and blocked in 5% skimmed milk diluted in TBS-T (20 mM Tris pH 7.4, 120 mM NaCl and 0.05% of Tween 20) and after 3 successive washings with TBS-T, membranes were incubated with sheep sera diluted at 1:1500 for 1 h over a stirrer at room temperature. After 3 washes with TBS-T, incubation with the secondary antibody conjugated to alkaline phosphatase diluted 1:7000 in TBS-T for 1 h was performed. Membranes were incubated in an NBT/BCIP chromogen solution in phosphate buffer for 15 min for staining. Scans of serum that was negative for *C. pseudotuberculosis* as well as empty (non-recombinant) bacteriophage λ Zap Express were used as negative controls. Immunopositive plaques resulted in small reddish dots on the membranes. These membranes were then aligned with the original agar plate, allowing identification of corresponding plaques that were removed from the plates with a security area (approximately 0.5 cm of diameter) using a Pasteur pipette. Fragments of soft agar containing the positive clones were separately placed in microcentrifuge tubes with 250 μl SM buffer. Each tube was vortexed to release particles of recombinant phage in SM buffer. The tubes containing phages were stored at −20 °C.

### PCR amplification of the cloned inserts in pBK-CMV phagemid vector and sequencing

DNA inserts from positive clones present in recombinant phage were amplified by PCR with the oligonucleotides from pBK-CMV: T7 (5′ – GTA ATA CGA CTC ACT ATA GGG C - 3′) and T3: (5′ - CCC TTT AGT GAG GGT TAA TT - 3 ‘), 0.2 mM dNTPs, Taq DNA polymerase buffer, 1.5 mM MgCl_2_ and 0.5 U Taq DNA polymerase. The reaction was subjected to a denaturation step at 94 °C for 2 min followed by 35 cycles of denaturation at 94 °C for 30 s, hybridization of primers at 54 °C for 30 s and extension at 72 °C for 4 min.

The amplified fragments were purified and subsequently cloned into pGEM-T easy vector (Promega®). Recombinant plasmids were inserted in *E. coli* DH5α for proliferation then purified using the Qiaprep Spin Miniprep (Qiagen®) kit. With purified samples, the sequencing reaction was performed with the Big Dye Terminator v3.1 kit (Applied Biosystems®). The samples were sequenced in a capillary sequencer (Applied Biosystems® model 3130).

### Data processing

The sequences of the recombinant clones were analyzed by comparison with GenBank sequences (Table 1) using the BLAST algorithm and the Bio Edit® and Vector NTI 11® softwares. The sequences were also translated into amino acid sequences thus generating a fragment of the corresponding protein. Translated protein sequences were then analyzed for size and molecular weight with the help of Bio Edit® and Vector NTI 11®.

**Table 1:** Genetic sequencing analysis by comparing of the positive clones of *C. pseudotuberculosis* with the GenBank

Clones	Genes	E-value^a^	Identity (%)	GenBank access	Region of the genome		Size	
					Sequenced fragment	Total gene	Sequenced fragment (bp)	Total gene (bp)
1	*dak2*	0.0	100	CP001829	980,881..981600	979,885..981600	720	1716
2	*fagA*	3e-84	100	AF401634	1918..2090	1917..2984	174	1068
	*fagB*	0.0	100	AF401634	1278..1917	931..1917	640	987
3	NlpC/P60 protein family	5e-76	100	CP001829	482,613..482770	482,613..483479	158	867
4	LPxTG putative protein family	0.0	100	CP001829	2,269,628..2270599	2,269,628..2271304	972	1677

^a^The closer to zero the E-value, the lower the probability of the alignment of the gene sequences occurring at random

## Results

### Construction and validation of gene expression *Corynebacterium pseudotuberculosis* library

The average size of inserts cloned in the library was 3 kb, which, in total, corresponds to 576.000 kb or 5.76 × 10^8^ bp recombinant phage. This means 10 times greater coverage of the genome. Following amplification steps, the title of the library was 7 × 10^8^ CFU/ml.

Validation was carried out by PCR using the entire library. The amplified product corresponds to 924 bp of the *pld* gene, confirming the presence of this gene in the gene expression library of *C. pseudotuberculosis* (data not shown).

### Immunoscreening of the *Corynebacterium pseudotuberculosis* gene expression library

**Fig. 1:**
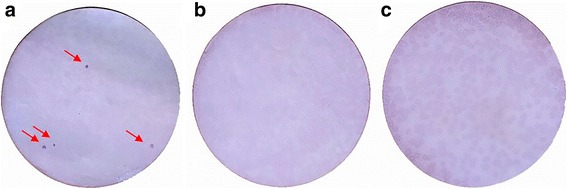
Immunoscreening. **a** Immunoscreening with positive sera highlighting four positive clones (red arrows). **b** Immunoscreening with negative control serum. **c** Control clones using λ Zap Express vector without insert

Four positive clones were identified in the immunoscreening. In contrast, both negative controls (negative animal serum and bacteriophage λ Zap Express without insert) showed no reactivity of the sera with the plaques (Fig. 1).

### Obtaining of the sequence of antigenic protein gene encoders for *Corynebacterium. pseudotuberculosis*

**Fig. 2:**
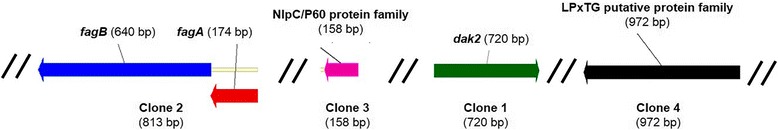
Genome order of *Corynebacterium pseudotuberculosis* genes identified by sequencing. Scheme representing the order of genes in the genome of *C. pseudotuberculosis* which were identified bysequencing based on immunoscreening reactive clones in the library. The amount of base pairs in brackets corresponds to the size of the fragment cloned and sequenced (not the total size of the gene). The parallel bars (in black) indicate the presence of more genes before, after and between the genes identified

The amplified fragments, with T7 and T3 primers by PCR of the four clones, were identified by sequencing and confirmed as belonging to the *C. pseudotuberculosis* genome (Table 1). The fragments of the genes identified in the sequences do not correspond to the total size of each gene, however, the actual size of each gene and the location in the bacterial genome can be seen on Table 1. According to location data from Table 1, the order of those genes in the genome of *C. pseudotuberculosis* is schematized in Fig. 2.

It was possible to align the sequences obtained from each gene with sequences from different strains of *C. pseudotuberculosis* using the Bio Edit software and in some cases sequences of genes from *Corynebacterium ulcerans* were also aligned. All sequences from different strains were obtained from GenBank. Sequenced fragments of *dak2* and *fagA* genes were shown to be conserved among different strains of *C. pseudotuberculosis*. The *fagB* gene and the LPxTG putative protein family gene are conserved among the different strains of *C. pseudotuberculosis*, however, they present differences in nucleotide sequences among three different strains of *C. ulcerans*. Only the NlpC/P60 family protein gene had well-conserved nucleotide sequences instrains of both *C. pseudotuberculosis* and *C. ulcerans*.

## Discussion

The advantage of building a library is that prior knowledge of target antibodies is not required, eliminating the need to design primer oligonucleotides and allowing the identification of new antigens. Moreover, with large part of the bacterium’s genome used in the library, it is possible to screen for the expression of several genes in the same experiment [18]. In the present study, immunoscreening of the gene expression library was efficient at finding genes that encode antigenic proteins of *C. pseudotuberculosis*.

The *dak2* gene encodes the dihydroxyacetone kinase 2 enzyme (DhaK2) and this protein reacted in clone 1 (Table 1). This enzyme converts dihydroxyacetone (DHA) into dihydroxyacetone phosphate (DHAP), because the kinases catalyze and transport ATP’s terminal phosphate to compounds with phosphate receptors [19, 20]. When converted to DHAP, the DHA has metabolic functions in animals, plants and bacteria. In bacteria, it participates in the glycolytic pathway and is an important phosphate source for energy generation [21, 22].

DhaK2 can also act in the evasion of the immune response in certain viruses. Dengue virus, like other RNA viruses, can benefit from the performance of this enzyme for disease development. DhaK2 acts by interrupting the retinoic acid I (RIG-I) inducer gene, which synthesizes a virus sensor protein and helps to modulate the immune response. With the interruption of this sensor, certain interferons are reduced, which compromises the antiviral response [23, 24].

Clone 2 had two purposes: to sequence genes and to compare them with genomic sequences in GenBank. This process led to the identification of iron transporter genes (Table 1). These genes are *fagA* and *fagB*, which are members of an operon that also includes genes *fagC* and *fagD*. This operon encodes membrane proteins, which are responsible for the uptake and transport of iron for the metabolism of *C. pseudotuberculosis*, as well as for other bacteria, such as *Salmonella enterica typhimurium* [2, 25]. In this experiment, however, we did not identify which of these two was the reagent or if both reacted to positive sera for CLA. Even with the partial identification of the two genes, both were considered as candidate antigenic genes by being part of the same operon.

One study reported that a mutant sample of *C. pseudotuberculosis* lacking the *fagB* gene was inoculated in goats for virulence tests. It did not cause lesions in the lymph nodes, and it was thus suggested that the bacteria did not develop in the animal organism due to a deceased ability to capture iron for their metabolism. This suggests that the *fagB* gene is associated with *C. pseudotuberculosis* virulence [2]. Studies on the bacterial genome revealed the organization and conservation of the *fag* operon between different samples of *C. pseudotuberculosis* and the affinity of the encoded proteins to bind and transport iron was confirmed by computational analysis [26, 27].

There are immune system compounds in mammals that act against enterobactin, the name given to bacterial siderophores (iron transporters). Lipocalin 2 binds to siderophores and activates the innate immune system against bacterial infection [28]. *Vibrio cholerae* was described as being able to evade the immune system because siderocalin does not bind to vibriobactin, which in turn does not activate the innate immune system of the host [29]. In this study, the *fagA* or *fagB* gene proteins were shown to be antigenic after reacting with sera from animals positive for CLA. They therefore become possible candidates for the development of serological diagnostic tests, as well as for the development of a vaccine against CLA. We cannot rule out the possibility of other operon proteins having antigenic potential since they are arranged on the C. *pseudotuberculosis* membrane.

The gene that encodes a hypothetical protein from the NlpC/P60 family, identified in clone 3 (Table 1), has been previously described in another work whose objective was to map the genome of different samples of *C. pseudotuberculosis* [26, 30]. The NlpC/P60 protein family are found in prokaryotes, eukaryotes and viruses [31]. They have protease, amidase or acyltransferase activity and are involved in the hydrolysis of cell wall peptides as well as in cell division [32–35]. Different bacterial proteins from this family have been compared and it has been reported that they have conserved residues or regions but it is not possible to determine if these regions are related to the infectivity of the pathogen [36].

In *Mycobacterium avium paratuberculosis*, which causes Johne’s disease in ruminants, the NlpC/P60 protein, encoded by the MAP1272c gene, reacted with bovine serum positive for the disease [37]. Considering the results of MAP1272c gene protein of *M. avium paratuberculosis* and knowing that *C. pseudotuberculosis* is part of the same suborder (*Corynebacterineae*), it can be hypothesized that these proteins have antigenic properties in different pathogens.

In another study, the analysis of the Rv2190c gene from *Mycobacterium tuberculosis*, also belonging to the *Corynebacterinae* suborder, revealed that the encoded protein also reacted to rabbit serum that was positive for infection of this bacterium. In the same study it was proven that the protein participates in the growth and formation of the bacterial cell wall [38]. A study on the G6R protein of the vaccinia virus has demonstrated that it participates in the virus-host relationship, being important in the establishment and development of virus infectivity in the vertebrate host [39]. This protein may have antigenic properties, as reported in bacteria of the *Corynebacterinae* suborder, which would favor the diagnosis of the disease caused by this or other pox viruses since these viruses have genes that encode this type of protein and are well conserved within the group [40, 41].

Clone 4, which reacted with sera from positive animals, has a gene that encodes a putative protein from the LPxTG family. The sequence of this gene has been previously described in a study of the *C. pseudotuberculosis* genome [42]. Proteins of this type are anchored to the cell wall of Gram-positive bacteria, such as *C. pseudotuberculosis*, by a sequence motif of amino acids specific to this group of bacteria. The LPxTG sequence motif is composed of the amino acids leucine, proline, x = any amino acid, threonine and glycine. The LPxTG motif is responsible for connecting the peptidoglycan, which forms the thick cell wall of Gram-positive bacteria [43].

The enzyme cysteine protease-transpeptidase sortase A (SRTA) or sortase A, cleaves the LPxTG motif between the glycine and threonine residues and covalently binds protein membranes to the same site. The proteins that bond to the LPxTG motif are usually related to the virulence of the bacteria since they assist in the adhesion and invasion of host cells [44]. A study carried out with *Streptococcus gordonii* showed that different LPxTG family proteins exist in the same bacteria, which have a conserved sequence of this motif. These proteins in *S. gordonii* were identified as adhesins [45]. In sortase A deleted mutants of *Listeria monocytogenes*, virulence was reduced when compared to wild strains. Bacteria was shown to be unable to enter the host cell. Without sortase A, adhesion proteins were not covalently bound to LPxTG, leading to this result [46].

The PfbA protein of *Streptococcus pneumoniae* was identified as an LPxTG family protein that assists in bacterial adhesion and invasion in human lung cells. It was reported that when *S. pnemoniae* underwent growth in human blood containing anti-PfbA antibodies, there was a 50% reduction in growth when compared to that without antibodies. This leads to believe that this protein triggers an immune response with the bacteria, making it a candidate as a vaccine against the disease [47]. In this study, the LPxTG protein family identified by comparison of genomic sequences in GenBank was shown to have antigenic characteristics in *C. pseudotuberculosis* just as in *S. pneumonia,* however, there is no evidence of the actual function of this protein in *C. pseudotuberculosis*. One can hypothesize that it is an adhesin and favors the development of CLA.

Genes reported herein encode membrane proteins. Proteins of this type can be antigenic, favoring the development of serological tests for diagnosis as well as the development of vaccines against CLA. This is enhanced when analyzing the alignment of each gene with the sequences of other strains of *C. pseudotuberculosis* in GenBank. If these genes and their proteins are conserved among different strains of bacteria, theoretically a vaccine or a diagnostic test developed with them would be effective against CLA caused by different strains in different regions. The gene from the NlpC/P60 protein family, which was well-conserved among different strains of *C. ulcerans,* however, may have cross-reactivity between *C. pseudotuberculosis* and *C. ulcerans* if used in a diagnostic test against CLA.

## Conclusion

Immunoscreening of a gene expression library enabled us to work directly with *C. pseudotuberculosis* and to find its genes that reacted with sera from sheep carrying CLA. This technique is a viable alternative for laboratories that need to perform genetic analysis and determine the function of genes but are not equipped with bioinformatics devices. The genes found herein will be assessed in a new stage in order to determine if they could be potential antigens in immunological tests for CLA, such as ELISA and rapid tests such as that used in bovine tuberculinization, as well as in vaccine formulations against *C. pseudotuberculosis*.
